# Magnetic Control of Nonmagnetic Living Organisms

**DOI:** 10.1021/acsami.4c02325

**Published:** 2024-03-26

**Authors:** Ahmed Al Harraq, Min Feng, Hashir M. Gauri, Ram Devireddy, Ankur Gupta, Qing Sun, Bhuvnesh Bharti

**Affiliations:** † Cain Department of Chemical Engineering, Louisiana State University, Baton Rouge, Louisiana 70803, United States; ‡ McFerrin Department of Chemical Engineering, Texas A&M University, College Station, Texas 77843, United States; § Department of Mechanical and Industrial Engineering, Louisiana State University, Baton Rouge, Louisiana 70803, United States; ∥ Department of Chemical and Biological Engineering, University of Colorado, Boulder, Colorado 80303, United States

**Keywords:** magnetophoresis, magnetostatics, active matter, biohybrids, microrobotics, magnetic manipulation

## Abstract

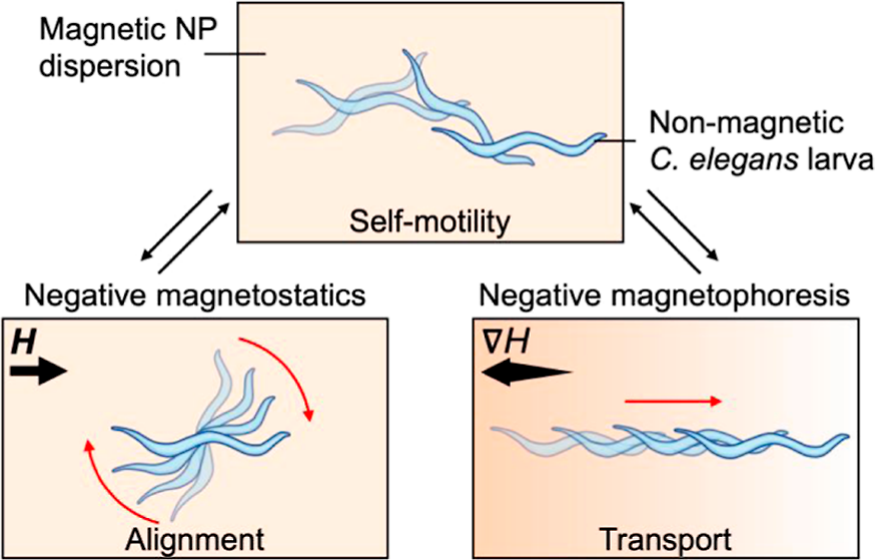

Living organisms inspire the design of microrobots, but their functionality is unmatched. Next-generation microrobots aim to leverage the sensing and communication abilities of organisms through magnetic hybridization, attaching magnetic particles to them for external control. However, the protocols used for magnetic hybridization are morphology specific and are not generalizable. We propose an alternative approach that leverages the principles of negative magnetostatics and magnetophoresis to control nonmagnetic organisms with external magnetic fields. To do this, we disperse model organisms in dispersions of Fe_3_O_4_ nanoparticles and expose them to either uniform or gradient magnetic fields. In uniform magnetic fields, living organisms align with the field due to external torque, while gradient magnetic fields generate a negative magnetophoretic force, pushing objects away from external magnets. The magnetic fields enable controlling the position and orientation of *Caenorhabditis elegans* larvae and flagellated bacteria through directional interactions and magnitude. This control is diminished in live spermatozoa and adult *C. elegans* due to stronger internal biological activity, i.e., force/torque. Our study presents a method for spatiotemporal organization of living organisms without requiring magnetic hybridization, opening the way for the development of controllable living microbiorobots.

## 1 Introduction

Recent decades have seen rapid growth in the field of robotics, with increasingly autonomous robots even inside households. However, a notable lag exists in the development of robots that can operate autonomously and perform complex functions at the nano- and micron scales.^1^ This lag primarily stems from the challenge of not being able to simply “shrink” or miniaturize traditional robots to the micron scale without compromising their functionality. Instead, emerging bottom-up strategies are being developed that prioritize the utilization of active colloidal particles^2^ capable of sensing and responding to external stimuli with programmable motility,^3,4^ although they often lack the comprehensive functional aspects of traditional robots.^5^ While inspired by biological matter,^6^ these synthetic active materials fall short of the hierarchical complexity found in living colloids.^7^ In stark contrast, organisms ranging from single-celled microbes to small animals represent an ultrafunctional form of active matter, showcasing true autonomous operation at small length scales. Hence, nature provides a diverse range of components for the design of “living devices” with varying degrees of cognition and capability to perform biological functions. While the interest in exploiting the functional advantages of biological colloids is growing,^8−10^ microrobotics based on living organisms faces a grand challenge—the need for a versatile tool for the precise spatial manipulation of nonmagnetic living organisms.

Magnetic fields represent a versatile method to control organisms in a contactless and chemically inert fashion and are highly effective at length scales as small as a few nanometers.^11,12^ However, since most organisms do not display significant magnetizability, recent reports have devised techniques for magnetic hybridization.^13−15^ These involve embedding a magnetic domain onto the organism, allowing indirect manipulation with an external magnetic field.^16^ Magnetic biohybrid microrobots show much promise in protocols that require cell delivery such as in vitro fertilization^17^ and biomedical applications.^18^ Most hybridization methods are heavily reliant on the physicochemical characteristics of the organism(s) and raise concerns regarding their viability.^9^ In addition, hybridization often involves neutralization of the organism, limiting its utility purely to its soft body rather than its biological function.^19,20^ At present, there are no available techniques that allow for the magnetic manipulation of nonmagnetic living organisms without requiring targeted interactions, particle internalization, or shape-specific “shuttles”.

**Figure 1:**
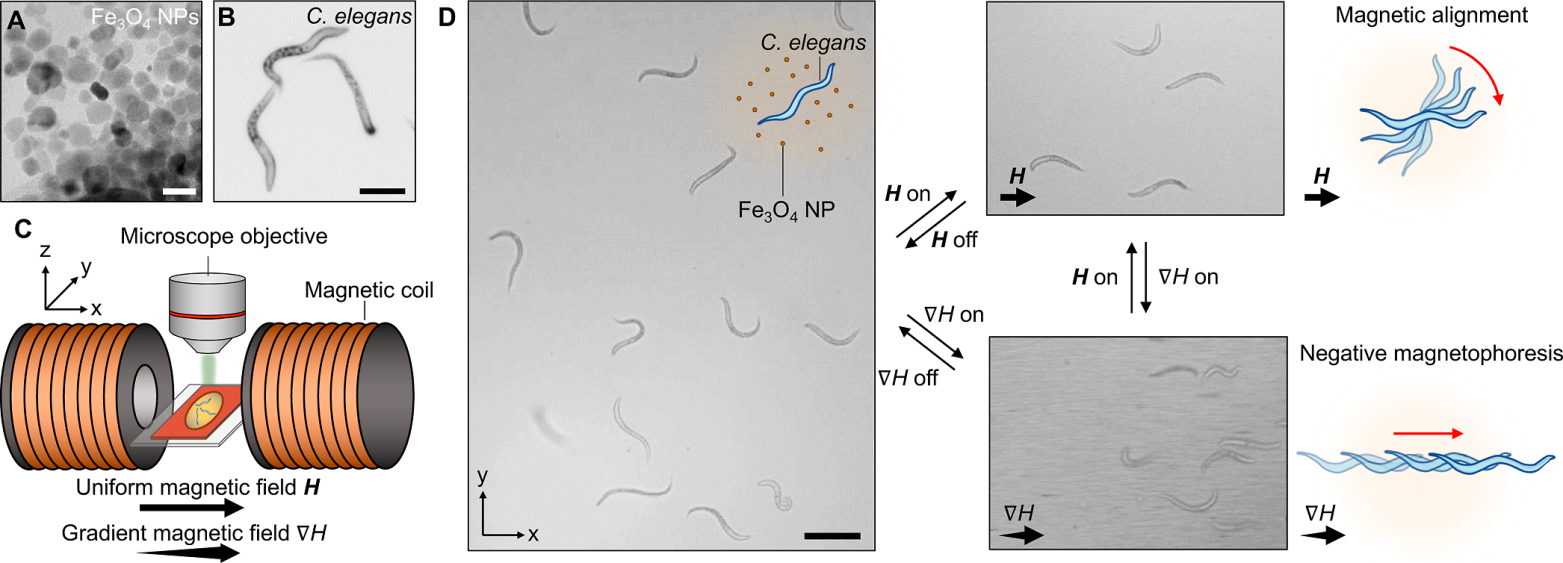
Application of magnetic fields on *C. elegans* nematodes dispersed in magnetic nanoparticle dispersions. (A) Transmission electron micrograph of iron oxide nanoparticles (Fe_3_O_4_ NPs) composing the ferrofluid in use. Scale bar: 30 nm. (B) Optical microscopy image of *C*. elegans nematode worms selected as the main model organism for the study. Scale bar: 50 μm. (C) Schematic of custom Helmholtz coil setup used to induce either a uniform or a gradient magnetic field of tunable strength in the *x*-direction. (D) Microscopy images and corresponding schematics of freely swimming worms in the absence of field (left panel) aligning with the uniform magnetic field, ***H*** (top right panel), and repelled from regions of high magnetic field in a gradient, **∇***H* (bottom right panel). Scale bar: 100 μm.

We propose utilizing a dispersion of magnetically responsive nanoparticles (NPs) to manipulate nonmagnetic microorganisms through negative magnetostatics and magnetophoresis.^21,22^ In this approach, living organisms are introduced into an aqueous dispersion of iron oxide (Fe_3_O_4_) NPs of nominal radius ∼10 nm (Figure 1A). Given that the typical size of living organisms, such as bacteria and worms, is at least 2 orders of magnitude larger than the NPs, the dispersion can be treated as a continuum fluid in a uniform magnetic field. When subjected to an external magnetic field, the living organisms acquire a moment that scales as follows^23,24^
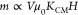
1
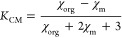
2where *V* is the volume of the organism, μ_0_ is the magnetic permeability, and ***H*** is the external field. *K*_CM_ is the real part of the Clausius–Mossotti function, where χ_org_ and χ_m_ are the magnetic susceptibility of the organism and the NP dispersion, respectively. In this approach, small living organisms with χ_org_ ∼ 0 are suspended in a magnetic NP dispersion with positive bulk magnetic susceptibility, χ_m_ > 0. The negative contrast in the susceptibility endows diamagnetic behavior to nonmagnetic organisms within the magnetic fluid (eq 2). Consequently, when exposed to an external magnetic field, microorganisms suspended in Fe_3_O_4_ NP dispersions acquire a dipole moment antiparallel to the field.^25−27^ We explore the application of this approach to regulate the motion and spatial distribution of nonmagnetic wild-type *Caenorhabditis elegans* (*C. elegans*) (Figure 1B) suspended in the magnetic NP dispersions and exposed to external magnetic fields. Specifically, we first demonstrate the use of our approach in conjunction with a uniform magnetic field and corresponding negative magnetostatics to control the orientation and thus the direction of the swimming of microorganisms. Second, we show how nonuniform magnetic fields allow controlling the position of the living worms through negative magnetophoresis. The article presents an approach of using uniform and gradient magnetic fields to control orientation and spatial distribution of living organisms in their nonhybridized state and discusses the potential for impact and shortcomings of the methodology.

## 2 Results and Discussion

### 2.1 Experimental Procedure

*C. elegans* is a common nematode worm that displays crawling motility on surfaces and swimming motility in bulk fluids.^28^ The developmental cycle of *C. elegans* involves four larval stages L1–4, followed by a young adult stage and the egg-laying adult stage^29^ (Figure S1). We dispersed *C. elegans* at a desired growth stage in commercially available aqueous ferrofluid EMG 705 (Ferrotec Inc.) containing 0.5% Fe_3_O_4_ NPs by volume, which are stabilized by proprietary anionic surfactants. A diluted suspension of this NP dispersion minimizes its toxicity to organisms within the time frame of experiments (typically a few hours). Alternatively, the analogous commercially available ferrofluid PBG 100 (Ferrotec Inc.) is a valid alternative rendered biocompatible by replacing surfactant with PEG coating of the NPs, where no significant change in the motility of worms is observed after 24 h of NP exposure (Figure S2). In a typical experiment, we sandwiched a 20 μL droplet of the NP dispersion containing *C. elegans* between a glass slide and a microscope coverslip separated by a 500 μm-thick silicone spacer. The sample was placed in a custom Helmholtz coil setup composed of two iron-core copper wire coils that were connected in series to a DC power supply (BK Precision) and fit under the objective of a Leica DM6 upright microscope (Figure 1C). By precisely tuning the current in the coils, we generate associated magnetic fields of calibrated strength which we measure using a gaussmeter (AlphaLab Inc. GM2). When the same current runs through the colinear coils, we develop a quasi-uniform field, ***H***, in the region where the sample is located: such homogeneous magnetic field can act to align magnetized objects. Using a single coil generates a gradient magnetic field, **∇***H*, analogous to using an external permanent magnet, which can cause regular magnetophoresis of paramagnetic objects or negative magnetophoresis of diamagnetic objects.^23^ Sample microscopy images and schematics of the effect of homogeneous and gradient magnetic fields on *C. elegans* are shown in Figure 1D.

### 2.2 Orientation Control by Uniform Magnetic Fields: Negative Magnetostatics

**Figure 2:**
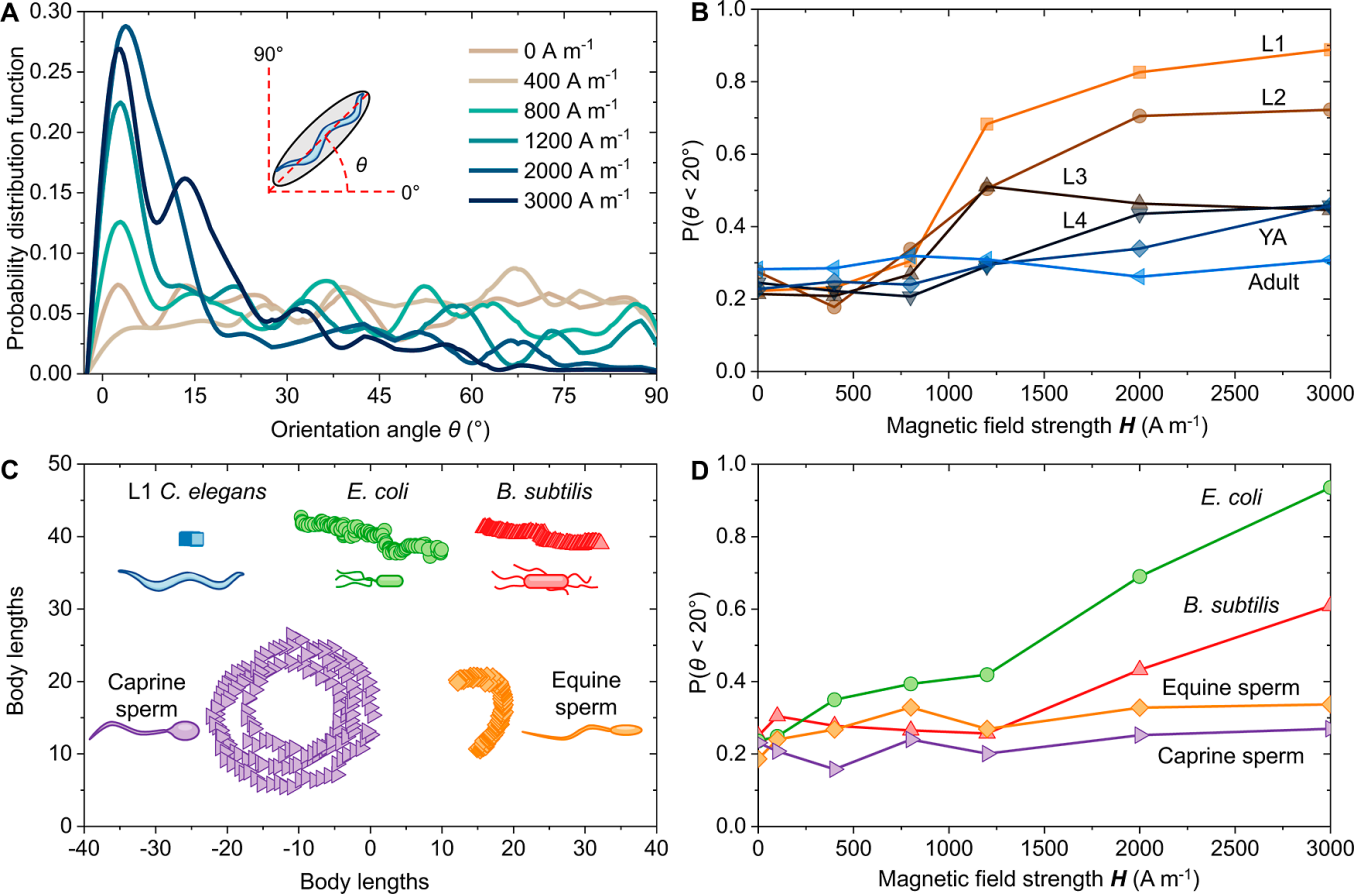
Regulating orientation of live organisms in uniform magnetic fields. (A) Distribution of the orientation angle, θ, obtained from ∼30 s recordings of *C. elegans* at the L2 stage swimming in Fe_3_O_4_ NP suspensions in uniform magnetic fields of varying strength. The orientation angle, θ, is defined as the angle between the major axis of the ellipsoid circumscribing the worm and the field direction at the 0° angle, as shown in the inset. The broad distribution of orientations observed when ***H*** = 0 is narrowed toward θ = 0° when the field is turned on. (B) Probability of θ being smaller than 20° to represent the degree of reorientation of worms of varying developmental stage in magnetic fields of increasing strength. L1 larvae are easily oriented by the magnetic field-induced torque, but they are less aligned as they grow until they are unaffected by the external field in their adult stages. (C) Tracked motion of organisms swimming for 15 s in an external magnetic field of strength ***H*** = 3000 A m^–1^. *E. coli* and *B. subtilis*, analogously to L1 *C. elegans*, orient their swimming in the *x*-direction, while spermatozoa move in curvilinear trajectories irrespective of the external field. (D) Probability of θ being smaller than 20° for the different organisms, analogous to the measurement in (B) showing the ability to orient the swimming motion of *E. coli* and *B. subtilis* with increasing ***H***, but not of equine and caprine spermatozoa.

The motion of *C. elegans* in bulk liquids is characterized by an undulatory swimming gait that normally occurs in response to external stimuli such as food availability, temperature, or the presence of other organisms.^29^ In the absence of such environmental triggers, *C. elegans* either rests in place or swims in seemingly random orientation due to the lack of global potential gradients. We test the effect of uniform magnetic fields to direct the orientation of the worms in equivalent force-free conditions. To do this, we position the magnetic NP dispersion containing worms in the center of the dual-coil setup, ensuring that the applied field, ***H***, is uniform. We record the motion of worms in the presence of uniform magnetic fields ***H*** of varying strength and measure the orientation angle, θ, of the worms. Here, we define θ as the angle between the major axis of the ellipsoid circumscribing the worm and the field direction as shown in the inset to Figure 2A. We extract θ over 30 s of swimming (2 fps) for 5 worms for each developmental stage under magnetic fields of varying strength. A typical data set for L2 worms shows that, before applying any magnetic field (i.e., when ***H*** = 0 A m^–1^), worms swim in random orientations (Figure S3). On the other hand, when we introduce a uniform magnetic field of strength ***H*** = 3000 A m^–1^, the worms align their bodies with the field closer to the 0° orientation (Video S1). Magnetically oriented worms swim with their own force in either of the two directions along the field axis, highlighting that ***H*** acts to align their bodies rather than imposing a global gradient.

Inducing a magnetic field through a DC powered electromagnet enables us to precisely manipulate the strength of the magnetic field to test the alignment of worms with increasing magnetic torque. We collapse all measurements of θ to the [0°, 90°] quadrant where θ = 0° corresponds to the field axis, and we extract the probability distribution of the swimming orientation of the worms (Figure 2A). For L2 larvae, ***H*** < 800 A m^–1^ is insufficient to achieve control over θ as the orientation of worms remains randomly distributed. When ***H*** ≥ 800 A m^–1^, the distribution of worm orientations becomes skewed toward low θ, highlighting a bias in the direction of motion. Ramping ***H*** up to 3000 A m^–1^ increases the probability of observing worms with θ < 20°, indicating a strong preference in their direction of motion along the field axis. We observe an increasing control of θ by increasing ***H*** only for *C. elegans* in their larval stages. This is particularly evident for L1 and L2 worms which display a cumulative probability of orientation near the field axis, *P*(θ < 20°), up to, respectively, 0.9 and 0.7 at ***H*** = 3000 A m^–1^. L3 and L4 larvae display a bias in their preferred orientation at high ***H*** with *P*(θ < 20°) = 0.4, while worms in their preadult stage show a limited skew of *P*(θ < 20°) = 0.3. Fully grown *C. elegans* are not affected by ***H*** up to 3000 A m^–1^. Note that the efficacy of the uniform magnetic field to align objects tends to plateau near 3000 A m^–1^ and decreases with a further increase in ***H*** (Figure S4). This is because stronger magnetic fields cause chaining of the Fe_3_O_4_ NPs and a subsequent loss in the net magnetic susceptibility of the fluid. At constant ***H*** in the 0–3000 A m^–1^, we find that adult worms are less susceptible to torque-driven magnetic manipulation in comparison to larvae (Figure 2B). This result is in contrast with the scaling of magnetic torque ***T***_**mag**_ with the size of worms approximated to prolate ellipsoids as follows^30^
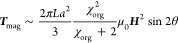
3where *L* and *a* are the length and diameter of the worm, respectively, and μ_0_ is the permeability of free space (=4π × 10^–7^ N A^–2^). As ***T***_**mag**_ increases with the worm size, so does the net internal torque, ***T***_**int**_, exerted by the worm to swim and change direction. Given that magnetic alignment of *C. elegans* occurs when ***T***_**mag**_ > ***T***_**int**_, our experiments indicate that ***T***_**int**_ scales faster with worm growth than ***T***_**mag**_. As a result, torque-driven alignment of *C. elegans* in magnetic fluids exhibits a stronger influence on young larvae which exert lower internal torques to swim.

The main advantage of diamagnetic manipulation in magnetic fluids is the ability to control virtually any dispersed organism, irrespective of its inherent magnetizability. To explore this feature, we set out to test the torque-driven alignment of a set of microorganisms of interest for hybrid microrobotics:^31^*Escherichia coli* (*E. coli*) and *Bacillus subtilis* (*B. subtilis*) flagellated bacteria and live equine and caprine spermatozoa (Table S1). We perform experiments analogous to those described for *C. elegans* by dispersing each organism in Fe_3_O_4_ NP dispersions and exposing them to uniform magnetic fields. We track the motion of organisms as shown in Figure 2C and extract θ as the angle between the major axis of the ellipsoid fitting the swimmer’s body and the axis of the magnetic field. In the absence of the field, all organisms move in random orientation, with the bacteria undergoing run-and-tumble motion and the spermatozoa progressing via undulation of the flagellum. We find that the orientation of bacteria, *E. coli* in particular, aligns with the field when ***H*** > 1200 A m^–1^. Conversely, the swimming of spermatozoa appears unaffected by the exposure to the magnetic field with motion in a random orientation for all ***H*** (Figure 2D). These findings further highlight that magnetic torque-driven alignment of living organisms depends on a competition between ***T***_**mag**_ and ***T***_**int**_ that varies across species. In fact, we have the highest *P*(θ < 20°) for *C. elegans* larvae in the 200–400 μm length range and for 1–2 μm long bacteria. Spermatozoa ranging from 5 to 10 μm in length do not respond to the externally imposed magnetic torque. The effectiveness of the field in controlling large worms and small bacteria but not intermediate-sized spermatozoa further highlights that the scaling of ***T***_**mag**_ with *L* is an insufficient predictor of alignment. Externally applied uniform magnetic fields correspond to a wall-like constriction acting locally with respect to the organism. Each organism either swims along or through this “invisible boundary” depending on a balance between ***T***_**mag**_ and the strength of its internal activity endowing ***T***_**int**_. By testing various microorganisms, we find that this balance likely varies across species due to differences in their modes of motion, from muscle-driven undulation of *C. elegans*(32) to helical rotation of bacterial flagella,^33^ to the two-dimensional beating of sperm flagella.^34^ Thus, a more predictive understanding of the applicability of diamagnetic manipulation of organisms in magnetic fluids must account for the various biophysical mechanisms of torque generation.

### 2.3 Spatiotemporal Control by Nonuniform Magnetic Fields: Negative Magnetophoresis

**Figure 3:**
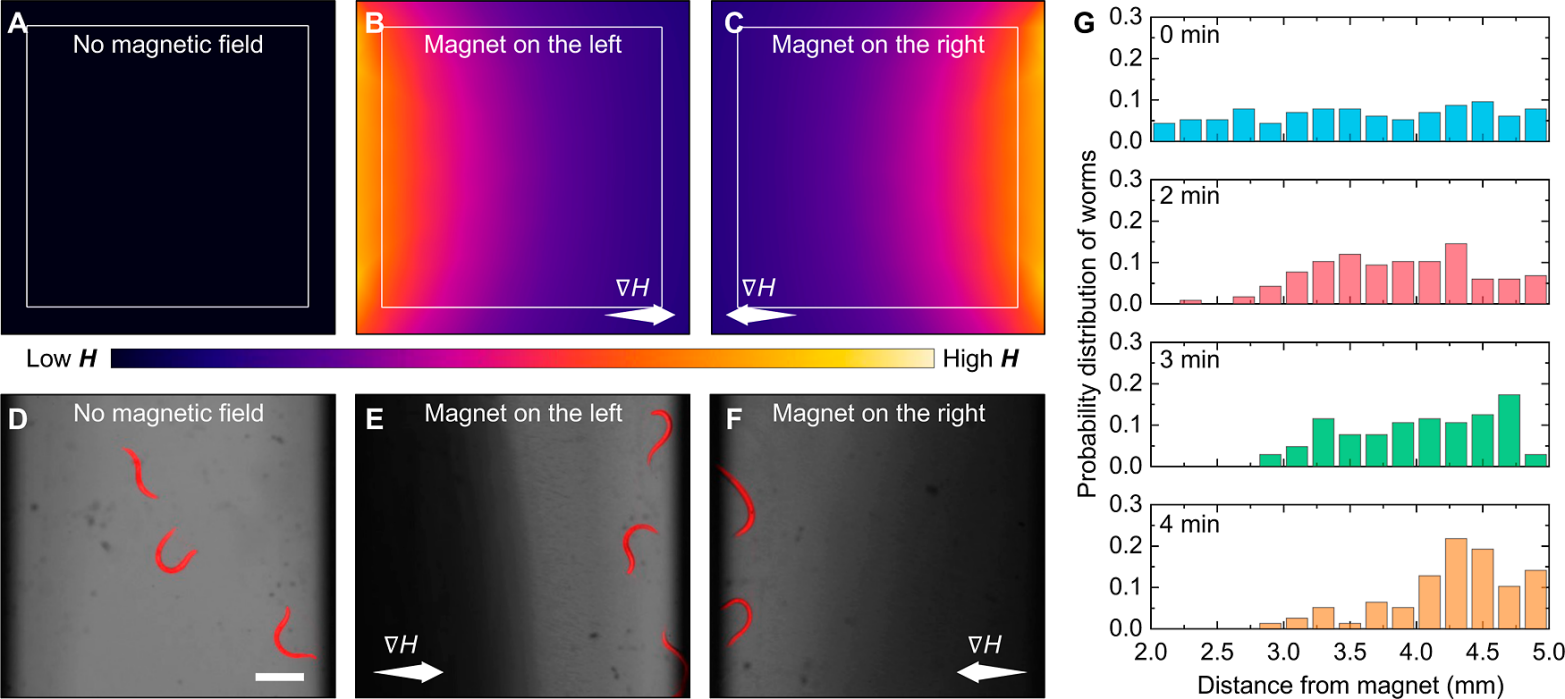
Negative magnetophoresis of *C. elegans* in gradient magnetic fields. (A–C) Finite element simulation of the magnetic field distribution (A) in the absence of magnetic field, (B) with a permanent magnet on the left-hand side, and (C) with a permanent magnet on the right-hand side. The numerical solution obtained using COMSOL Multiphysics shows the gradient from regions of low magnetic field (purple) to regions of high magnetic field (yellow). (D–F) Sample microscopy images of the negative magnetophoresis of *C. elegans* (false colored red) in Fe_3_O_4_ NP dispersion in response to gradient magnetic field of **∇***H* = 10^4^ kA m^–2^ across the chamber. Depending on the direction of the applied magnetic field gradients, the Fe_3_O_4_ NPs migrate toward the magnet as observed by the dark fluid area, while the worms travel in the opposite direction. Scale bar: 100 μm. (G) Initially uniform spatial distribution of *C. elegans* is controlled via negative magnetophoresis, as they concentrate in regions of low magnetic field.

In addition to torque-driven alignment, the effective magnetization of living organisms in bulk magnetic fluids enables force-driven manipulation in gradient magnetic fields. Magnetophoresis is the migration of objects *up* global gradients in the magnetic field, i.e., toward high field regions. Many applications rely on magnetophoresis of para- or ferromagnetic particles for separations such as in biological assays.^35^ On the other hand, diamagnetic objects experience negative magnetophoresis and migrate *down* global gradients in the magnetic field, i.e., toward low field regions.^36^ This is a much less common tool that is only recently gaining traction in areas of magnetic levitation and density-based analytical chemistry.^37^ The negative magnetophoretic force on a nonmagnetic object suspended in a magnetic medium is given as^19^
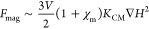
4All symbols are defined in reference to eqs 1 and 2. To test the effect of a gradient magnetic field, **∇***H*, on living nonmagnetic objects, we image a dispersion of *C. elegans* and Fe_3_O_4_ NPs in a 4.5 mm × 4.5 mm microfluidic chamber that is ∼80 μm thick. We quantify the local density of worms as we apply a gradient field using a 7.5 × 1.3 × 0.4 cm^3^ neodymium–iron–boron (NdFeB) bar magnet (KJ Magnetics) (Figure 3A). The permanent magnet applies a gradient field ranging from ∼60 kA m^–1^ to ∼14 kA m^–1^ across the 4.5 mm chamber, i.e., **∇***H* ∼ 10^4^ kA m^–2^, as measured experimentally (Figure S5). We simulate the distribution of magnetic field using COMSOL Multiphysics 5.3 to solve Gauss’ law based on the scalar magnetic potential and visualize the regions of high ***H*** and low ***H*** (Figure 3B, see Methods for details). Within the fluid area available for phoretic migration, ***H*** is highest near the magnet and lowest far from the magnet. The superparamagnetic Fe_3_O_4_ NPs will travel toward the magnet, while the *C. elegans* would be pushed away from the magnet via negative magnetophoresis. We record this **∇***H*-induced migration (Figure 3C–E) and track the position of each worm to quantify its density with the distance from the magnet. Worms that are originally uniformly distributed across the chamber migrate away from the magnet concentrating on one side of the chamber within 5 minutes (Figure 3E and Video S2).

**Figure 4:**
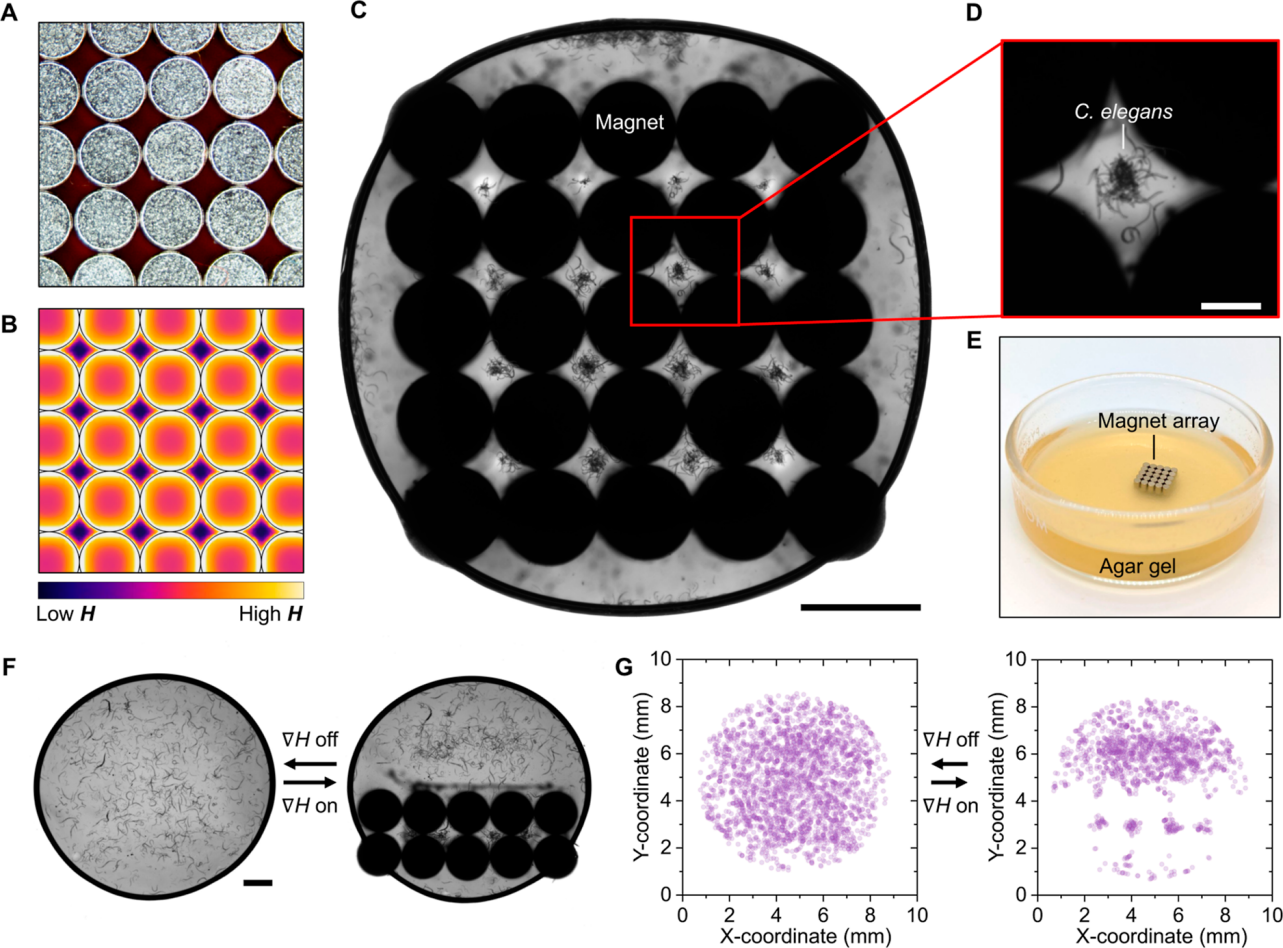
Spatiotemporal control of *C. elegans* using gradient magnetic fields. (A) Photograph of an array of NdFeB disk-shaped magnets (1.5 mm diameter) and (B) corresponding finite element simulation of the magnetic field distribution displaying the periodic arrangement of low and high magnetic field regions. The voids between four adjacent magnets represent areas of lowest magnetic field. (C) Microscopy image of a droplet of Fe_3_O_4_ NP dispersion containing *C. elegans* that are (D) confined via negative magnetophoresis in the regions between magnets corresponding to the magnetic field minima. Scale bar in (C): 2 mm. Scale bar in (D): 0.5 mm. (E) Photograph of the magnet array embedded in the agar plate for partitioning worms on the gel surface (as shown in Figure S6). (F) Microscopy images and (G) coordinate plots tracking the position of worms that switch from being randomly distributed in the liquid (left) to being localized in a 2 × 5 array of disk-shaped magnet. For all experiments presented in the figure, the field gradient between the surface of the disc-shaped magnet and the center of the void was **∇***H* ∼ 10^4^ kA m^–2^. Scale bar for (F): 1 mm.

Rendering small organisms diamagnetic enables their precise localization using simple NdFeB magnets. Arranging small magnets in arrays allows control over the distribution of magnetic field that drives magnetophoresis (Video S3). For example, placing disk-shaped magnets (1.5 mm diameter, KJ Magnetics) into a 5 × 5 square lattice gives rise to an associated 4 × 4 lattice of voids (Figure 4A). This arrangement is characterized by sharp gradients in the magnetic field separating regions of high ***H*** that correspond to the magnets and regions of low ***H*** corresponding to the voids, as shown via finite element simulation (Figure 4B). When placing a dispersion of *C. elegans* and Fe_3_O_4_ NPs, we observe rapid migration of worms toward the voids which act as localization sites (Figure 4C,D and Video S4). This negative magnetophoresis offers a versatile method to control the distribution of living microswimmers constrained in millimeter-sized areas by virtual magnetic walls. The approach offers high modularity in determining the number and position of the localization sites. In addition, the size of the sites is controlled by the packing efficiency of the magnets and can be tuned, for example, by using cubic or ring-shaped magnets. The strength of the magnetic constriction is governed by the sharpness of the gradient, which can be tuned by stacking multiple magnets. A major advantage of magnetic manipulation is its contactless nature, which lends itself to incorporating this technique onto existing devices and common substrates. For example, magnet arrays can be embedded into agar gel as shown in Figures 4E and Figure S6. Notably, this approach for spatial organization of living organisms can be applied dynamically: *C. elegans* are released from magnetic constrictions upon removal of the magnets (Figure 4F,G, Supporting Information, and Video S3). Therefore, **∇***H* is also easily tuned in time offering a versatile tool to control the position of worms as they navigate complex on-chip environments such as mazes (Figure S7).

## 3 Conclusions

Dispersion of *C. elegans* in stable suspensions of Fe_3_O_4_ NPs unlocks the toolset of magnetic fields for the control of worm alignment and spatial organization. The uniform applied field imposes a torque that can overcome the internal activity of a variety of organisms and reduce their degrees of freedom favoring motion along the field axis. Thus, relatively weak fields below 3000 A m^–1^ are reportedly sufficient to control the average alignment of young worms as well as flagellated bacteria. In addition, gradient magnetic fields enable the manipulation of the position of worms via negative magnetophoresis to localize worms within low magnetic field boundaries and aid their motion in complex environments. This is a versatile and modular methodology that can be leveraged with either electromagnets or permanent magnets. The increase in the availability of highly biocompatible ferrofluids combines well with the ease of application of the techniques. Without any need for cumbersome and species-dependent hybridization steps, this approach enables programmable control over living matter. In this context, our results indicate a pathway to unlock applications that deploy small organisms and microbes in “living devices”. These include applications in drug discovery and regenerative medicine,^38^ pathogen and viral sensing,^39,40^ and also cell and small organism surgery.^41,42^

## 4 Methods

### 4.1 Strains and Sample Preparation

Wild-type N2 *C. elegans* strain was obtained from the *Caenorhabditis* Genetics Center (CGC). The strain was maintained on nematode growth medium (NGM) plates seeded with standard laboratory food *E. coli* OP50 at room temperature. Synchronized L1 worms were prepared by bleaching gravid adults to isolate embryos that hatched in the M9 buffer. OP50 *E. coli* and *B. subtilis* were grown overnight at 37 °C on Luria–Bertani (LB) plates from glycerol stocks. Then, bacteria from single colony were inoculated in LB broth and grown overnight at 37 °C with 250 rpm shaking. As the food, 500 μL of the OP50 overnight culture was seeded onto a 110 mm NGM agar plate and dried at room temperature prior to the addition of *C. elegans*. The equine semen samples were obtained from 3 stallions housed at the LSU Veterinary School. The extracted samples were diluted to 400–600 × 10^6^ viable cells/mL in a Kenney extender and kept at 37 °C. The caprine semen samples were obtained at the LSU Reproductive Biology Center. The extracted samples were diluted to ∼500 × 10^6^ viable cells/mL in a Triladyl extender at 30 °C. All experiments involving spermatozoa were done within 9 h after the samples were collected from the LSU Veterinary School and LSU Reproductive Biology Center, respectively. The dispersions of Fe_3_O_4_ NPs are aqueous-based ferrofluids (Ferrotec EMG705 and PBG100) diluted to approximately 0.5 v %. The NPs in EMG705 and PBG100 are stabilized, respectively, by adsorption of anionic surfactant and polyethylene glycol and are subject to aggregation in solutions at high ionic strength. To prevent this, we resuspended the model organisms in deionized water before mixing them with the ferrofluids. Worm suspensions were obtained by flooding the top of the plates with the NP dispersion and recollecting it together with worms. Suspensions of bacteria and spermatozoa were prepared by diluting stocks of cells in the NP dispersion. Samples were prepared by sandwiching 20 μL of the dispersion with NPs between a glass slide and a coverslip using a 0.5 mm-thick silicone spacer.

### 4.2 Magnetic Fields

Torque-driven alignment experiments were carried out by placing the sample containing NPs and worms between two iron–core–copper coils in a Helmholtz-type coil setup. The coils were connected in series to a DC power generator to induce an associated constant magnetic field. The strength of the field was measured in space using a gaussmeter (AlphaLab Inc. GM2). To induce a magnetic torque for aligning the organisms, we placed the sample in the region with a quasi-uniform field. For negative magnetophoresis experiments, we used permanent magnets (KJ magnetics) as described above. Arrays of magnets were devised by placing each adjacent magnet with inverse polarity and placed either on a glass slide or embedded in agar gel for subsequent imaging.

### 4.3 Microfluidic Chamber

Microfluidic square chambers were fabricated by using a standard photolithography procedure. First, SU-8-2050 photoresist (Microchem) was spin-coated to a thickness of 100 μm onto a precleaned silicon wafer. After soft-baking, the wafer was transferred to a direct-write optical lithography machine (MicroWriter ML3 Pro, Durham Magneto Optics) to pattern a 4.5 × 4.5 mm square chamber with a depth of ∼80 μm depth. After postbaking and developing the substrate, a template is recovered for replica molding of the chamber with polydimethylsiloxane (PDMS). To obtain the microfluidic device, the mold is cast with a 10:1 of PDMS elastomer-to-curing agent ratio (Sylgard 184), followed by curing for 2 h at 70 °C. Following curing, the peeled off devices were treated with oxygen plasma to render their surfaces hydrophilic before experiments.

### 4.4 Microscopy and Image Analysis

All bright-field imaging and recording were done using a Leica DM6 upright microscope equipped with a DFC9000 GTC camera, and the objectives used were Leica ×4/0.12 and Leica ×1.25/0.04. The orientation angles of worms were extracted by fitting ellipses over the bodies of the organisms using ImageJ. This was done either automatically after thresholding or via manual tracing when the suspension opacity due to magneto-optic effects hampered the computer-aided thresholding. Orientation data was collapsed to the [0–90°] quadrant, and probability distributions were obtained with the OriginPro software. Stereomicroscopy was done using the Leica MZ16FA. Images of the Fe_3_O_4_ NPs were obtained using a JEOL-JEM2010 transmission electron microscope operated at 200 kV.

### 4.5 Finite Element Calculations

Simulations of the magnetic field around permanent magnets were carried out using the AC/DC module in COMSOL Multiphysics 5.3. The ‘Magnetic Fields, No Currents’ module was used to solve Gauss’s law for the magnetic field using the magnetic potential as the dependent variable based on the input relative permeability and magnetization of each domain. The magnetization of the permanent magnets and relative permeability of surrounding materials were specified in the “Magnetic Flux Conservation” node. For the 4 × 1 cm bar magnet, we used a magnetization of 800 kA m^–1^, and for the 1.5 mm diameter disk magnets, we used a magnetization of 100 kA m^–1^, which were obtained from manufacturer data. The relative permeability of the NP dispersion was set to 1.1 based on previous measurement of the ferrofluid magnetic susceptibility.^26^

## Supporting Information Available

Developmental stages of *C. elegans*, motility of worms in ferrofluid, orientation of *C. elegans* in magnetic fields represented on polar plots, magnetic dipolar interaction energy between NPs, quantification of magnetic field gradient around the bar magnet, *C. elegans* on Agar pads containing immobilized magnets, magnetically assisted navigation of worms through maze, and mean size and velocity of organisms used in the study (PDF)
Alignment of worms (MPG)
Uniform distribution of worms (MPG)
Arrangement of small magnets in arrays (MPG)
Rapid migration of worms (MPG)

## Supplementary Material

am4c02325_si_001.pdf

am4c02325_si_002.mpg

am4c02325_si_003.mpg

am4c02325_si_004.mpg

am4c02325_si_005.mpg
