# Supplementary material for: Magnetic Control of Nonmagnetic Living Organisms
Source: ACS Appl Mater Interfaces. 2024 Mar 26;16(14):17339–46. doi: 10.1021/acsami.4c02325 (PMC11009914; doi:10.1021/acsami.4c02325)
Supplement: Supplementary file 1 — am4c02325_si_001.pdf [file am4c02325_si_001.pdf]

Supporting Information

Magnetic control of non-magnetic living organisms

Ahmed Al Harraq[1,‡], Min Feng[2], Hashir M. Gauri[1], Ram Devireddy[3], Ankur Gupta[4], Qing Sun[2,*] and Bhuvnesh Bharti[1,*]

[1] *Cain Department of Chemical Engineering, Louisiana State University, Baton Rouge, LA 70803*
[2] *McFerrin Department of Chemical Engineering, Texas A&M University, College Station, TX 77843*
[3] *Department of Mechanical and Industrial Engineering, Louisiana State University, Baton Rouge, LA 70803*
[4] *Department of Chemical and Biological Engineering, University of Colorado, Boulder, CO, 80303*
[‡]*Present address: Center for the Physics of Biological Function, Princeton University, Princeton, NJ 08544, USA*

*Corresponding authors' email: sunqing@tamu.edu, bbharti@lsu.edu

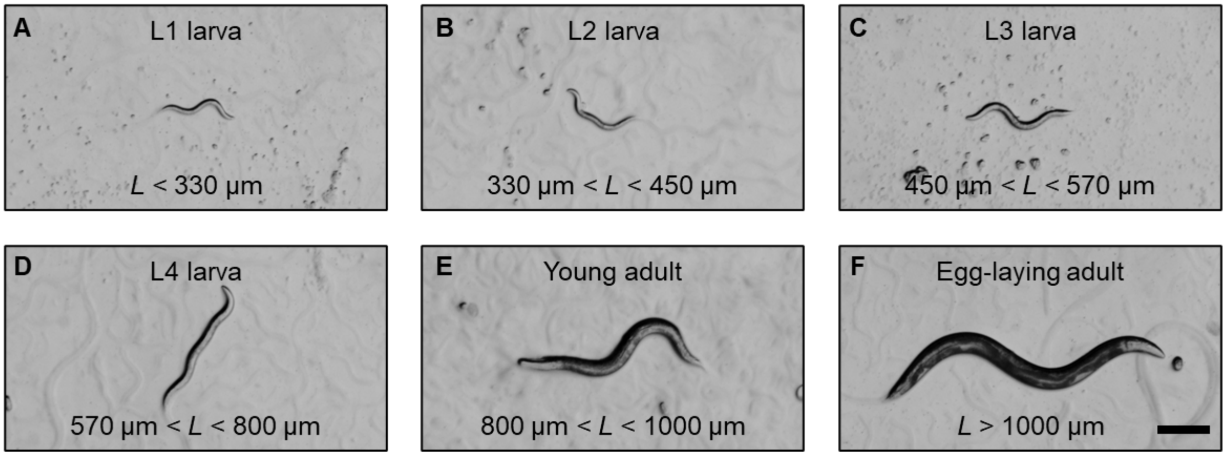

Figure S1. Sub-division of worm developmental stages. We classify worms within their traditional 6 stages, L1, L2, L3, L4, young adult, and adult, based on their length L. Worms having (A) $L < 330$ μm are L1 larvae; (B) 330 μm $< L <$ 450 μm are L2 larvae, (C) 450 μm $< L <$ 570 μm are L3 larvae, (D) 570 μm $< L <$ 800 μm are L4 larvae, (E) 800 μm $< L <$ 1000 μm are young adults, and (F) $L >$ 1000 μm are adults. Scale bar: 200 μm.

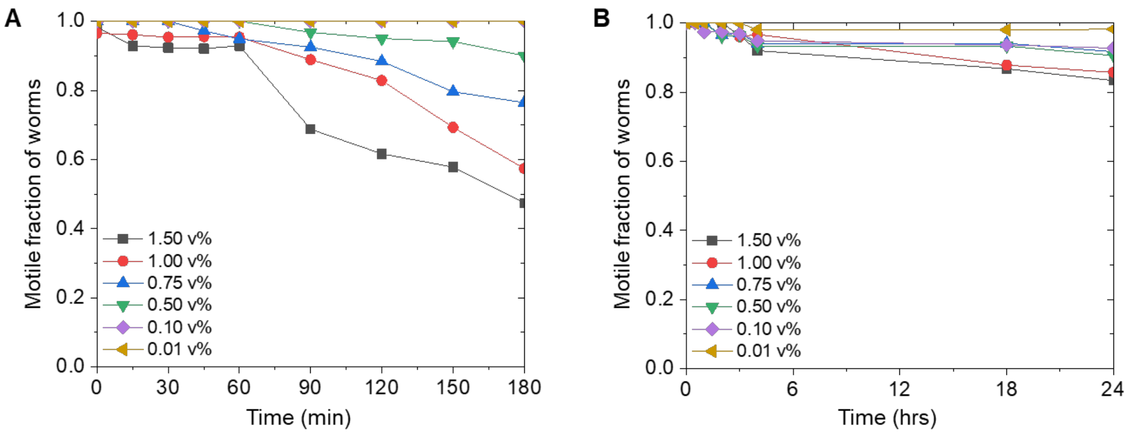

Figure S2: Fraction of motile worms in varying concentrations of Fe_3O_4 NP dispersions. (A) *C. elegans* worms quickly lose motility (observed via microscopy) when suspended in ferrofluids with more than 1 v% NP fraction. To prevent this, we limit the concentration of NPs to 0.5 v% in all experiments involving EMG 705. In addition, all experiments are done within 20 minutes of preparing the suspension. (B) PBG 100 is composed of NPs that are stabilized via a polyethylene glycol (PEG) coating, thus offering biocompatibility for days and at a wide concentration range.

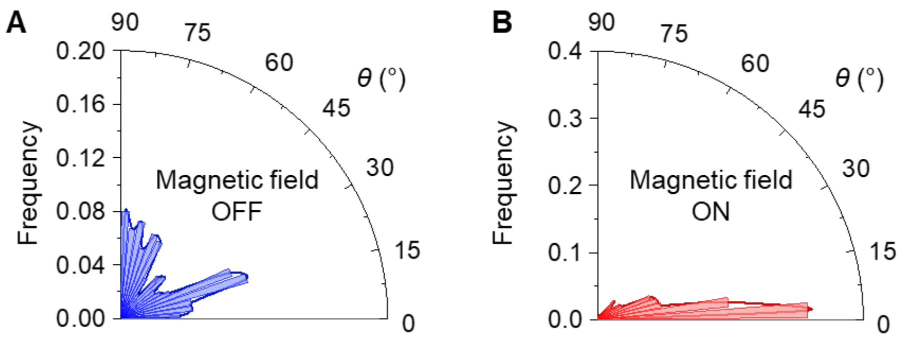

Figure S3. Polar plots of the orientation θ of *C. elegans* collapsed to the [0° 90°] quadrant obtained from ≥ 100 measurements. (A) In the absence of the magnetic field, the distribution of θ between the axes parallel ($\theta = 0°$) and orthogonal ($\theta = 90°$) to the direction of the magnetic field is uniform. (B) When $\boldsymbol{H} = 3000$ A m^{-1}, there is a narrowing of the distribution towards the external magnetic field direction highlighting the magnetic torque-driven alignment of the worms swimming orientation.

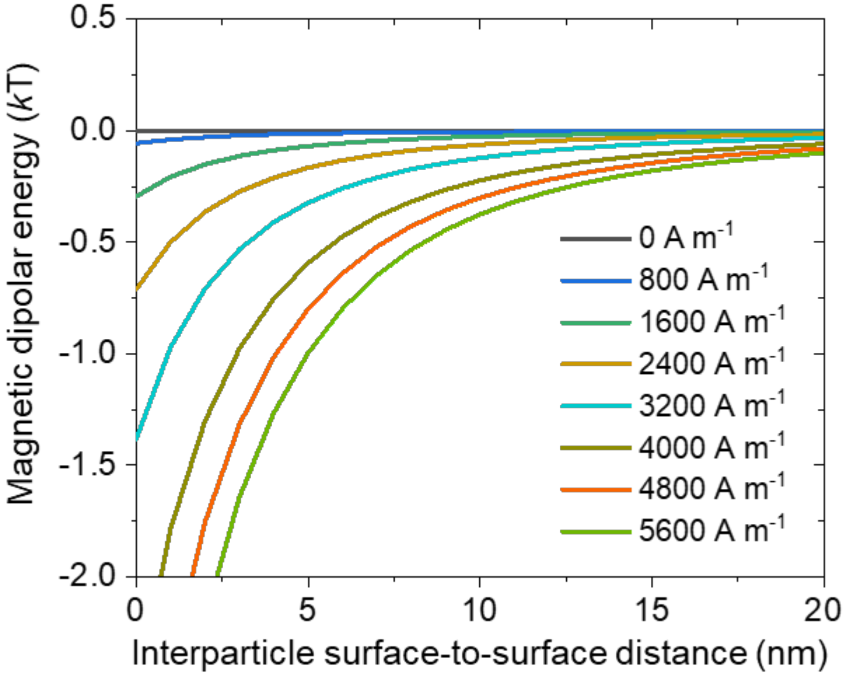

Figure S4. Magnetic dipolar interaction energy between Fe_3O_4 NPs in uniform magnetic field of varying strength $\boldsymbol{H}$. The magnetic interaction energy $\boldsymbol{U}_{mag}$ is obtained from $U_{mag} = \frac{\mu_0}{4\pi s}(1 - 3\boldsymbol{m}^2)$ where s is the interparticle distance and values of $\boldsymbol{m}$ at varying $\boldsymbol{H}$ are obtained experimentally from SQUID magnetometry[1]. It is observed that for $\boldsymbol{H}$ below approximately 3000 A m^{-1}, pairwise magnetic interaction are lower or in competition with thermal energy kT. Conversely, when $\boldsymbol{H} \geq 3000$ A m^{-1}, the associated magnetic dipolar interaction energy can lead to chaining which matches experimental observation.

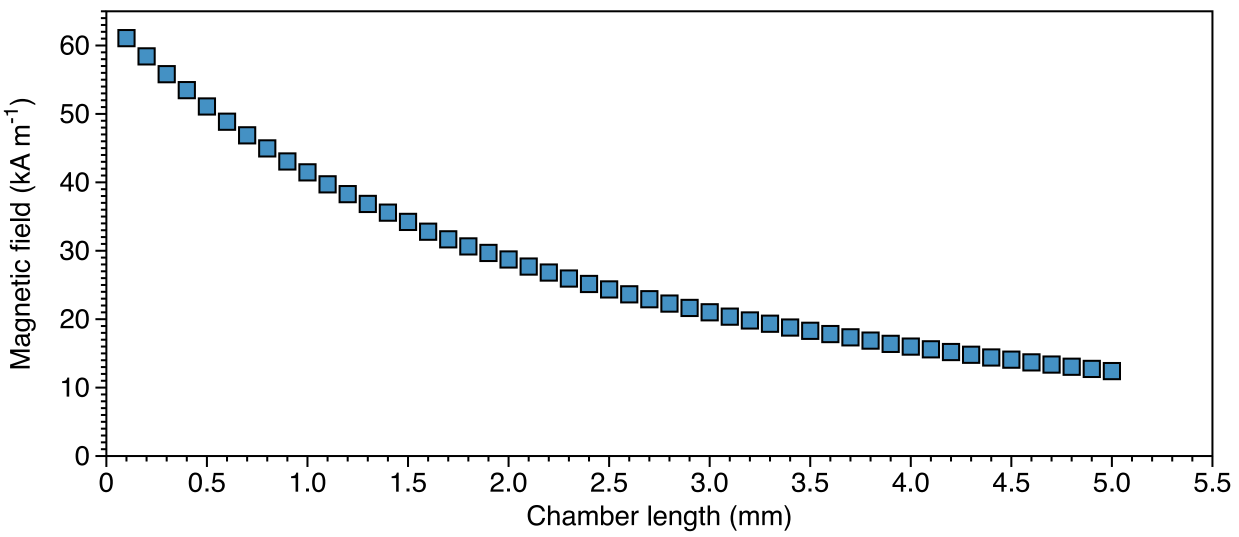

Figure S5. Magnetic field of bar magnet (7.5×1.3×0.4 cm^3; KJ Magnetics) measured across the length of the microfluidic chamber used in *C. elegans* inverse magnetophoretic experiments. The squares are gaussmeter readouts of the magnetic field.

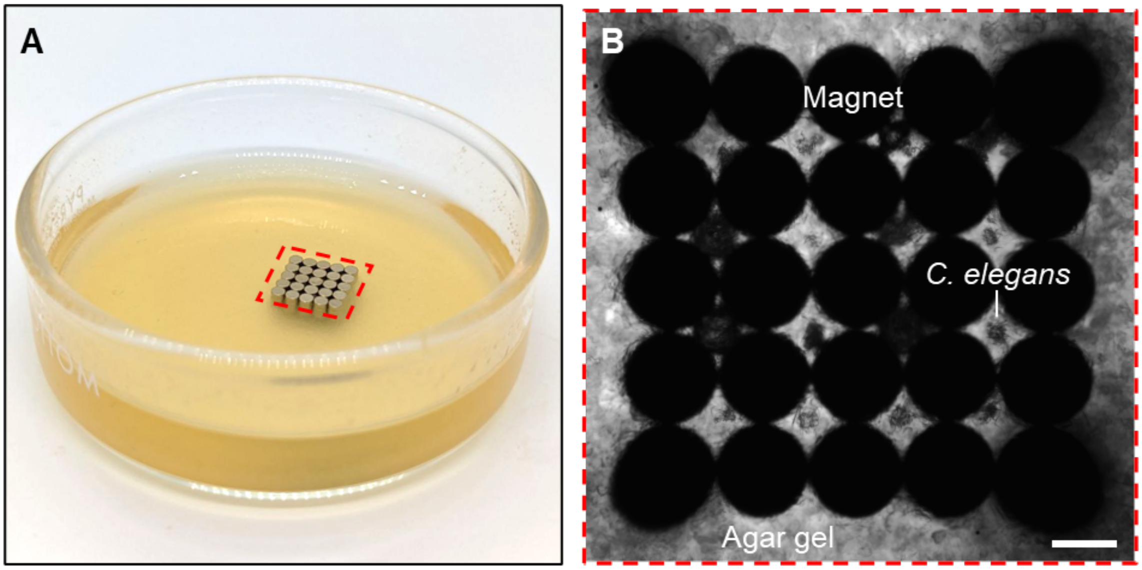

Figure S6. Magnetic localization of *C. elegans* on agar pad. (A) Disk-shaped magnets are embedded in the agar pad and (B) after dispersing worms in the suspension of Fe_3O_4 NPs, inverse magnetophoresis induced their localization in regions of lowest magnetic field, analogous to what shown in Figure 4. Scale bar: 1 mm.

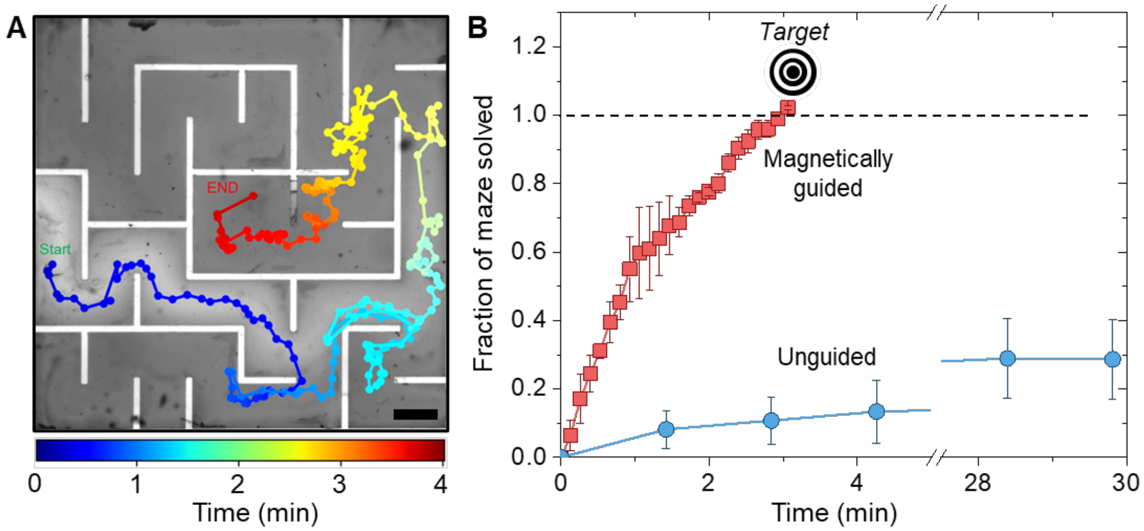

Figure S7. Magnetically assisted solution of a maze by *C. elegans.* (A) Maze constructed via traditional lithography with sample track of a worm solving the maze through magnetophoretic assistance using an external magnet. Scale bar: 500 µm.(B) Unguided worms are unable to solve the maze while magnetically guided worms can reach the end of the maze within a few minutes. Error bars represent the standard deviation from triplicate measurements.

Table S1. Mean size and velocity of model bacteria and spermatozoa in 0.5 v% EMG 705.

**Model organism**	**Mean size (length × diameter, µm)**	**Average velocity (µm s^{-1})**
E. coli	1.1 × 0.6	2
B. subtilis	3.2 × 1	18
Equine spermatozoa	6.3 × 2.1	159
Caprine spermatozoa	4.5 × 1.4	131

Captions to Supporting Information Videos

Supporting Information Video S1. Torque-driven alignment of *C. elegans* larvae upon application of uniform magnetic field $\boldsymbol{H}$ = 3000 A m^{-1}.

Supporting Information Video S2. Inverse magnetophoresis of *C. elegans* in Fe_3O_4 NP dispersion driven by a gradient magnetic field.

Supporting Information Video S3. Dynamic localization of worms in ‘magnetic voids’ formed via arrangement of disk-shaped magnets.

Supporting Information Video S4. Static localization of worms in ‘magnetic voids’ formed via arrangement of disk-shaped magnets.
